# Supplementary material for: Cometabolic Biodegradation of Hydrazine by Chlorella vulgaris–Bacillus Extremophilic Consortia: Synergistic Potential for Space and Industry
Source: Life (Basel). 2025 Jul 28;15(8):1197. doi: 10.3390/life15081197 (PMC12387905; doi:10.3390/life15081197)
Supplement: Supplementary file 1 [file life-15-01197-s001.zip › life-3758985-supplementary.pdf]

Supplementary Material

Cometabolic Biodegradation of Hydrazine by Chlorella vulgaris–Bacillus Extremophilic Consortia: Synergistic Potential for Space and Industry

Yael Kinel-Tahan[a], Reut Sorek-Abramovich[b], Rivka Alexander-Shani[b], Irit Shoval[c], Hagit Hauschner[c], Chen Corsia[a], Ariel Z. Kedar[a], Igor Derzy[d], Itsik Sapir[e], Yitzhak Mastai[f], Ashraf Al Ashhab[b,g], Yaron Yehoshua[a,*]

[a] The Algal Biotechnology Center, The Mina and Everard Goodman Faculty of Life Sciences, Bar-Ilan University, Ramat Gan 5290002, Israel. Email: yaelkinel@gmail.com; chenkorsia@gmail.com; arielkedar@gmail.com; yehoshy@biu.ac.il

[b] The Dead Sea & Arava Science Center (DSASC), Masada National Park, Mount Masada, Dead Sea 8691000, Israel. Email: reut.sorek@gmail.com; rivkasa@adssc.org, ashraf@adssc.org

[c] The Kanbar Core Facility Unit, The Goodman Faculty of Life Sciences, Bar-Ilan University, Ramat Gan 5290002, Israel. Email: irit.shoval@biu.ac.il; hagit.hauschner@biu.ac.il

[d] VTS Energy Ltd., Petah Tikva 4951939, Israel. Email: igor@vts-energy.com

[e] Department of Mechanical Engineering, Afeka Tel Aviv Academic College of Engineering, Tel-Aviv 6910717, Israel. Email: ItsikS@afeka.ac.il

[f] Department of Chemistry and Institute for Nanotechnology and Advanced Materials, Faculty of Exact Sciences, Bar-Ilan University, Ramat Gan 5290002, Israel. Email: mastai@biu.ac.il

[g] Ben-Gurion University of the Negev, Eilat Campus, Beer-Sheva 8410501, Israel. Email: ashraf@adssc.org

TABLES

Table S1: *C. vulgaris* growth with 0.5–10 ppb hydrazine measured by OD 680 nm.

No.	Hydrazine concentration (ppb)	Remarks
1	0.5	Experiment
2	1	"
3	2	"
4	5	"
5	10	"
6	0	Control – algae w/o hydrazine
7	0	Control – algae w/o hydrazine with titanium plate

Table S2: *C. vulgaris* growth with 50–1000 ppb hydrazine and control cultures (*n*=3).

No.	Hydrazine concentration (ppb)	Remarks
1	50	Experiment
2	100	"
3	500	"
4	1000	"
5	0	Control – algae w/o hydrazine
6	0	Control – algae w/o hydrazine with titanium

Table S3: *C. vulgaris* growth with 0.1–10 ppm hydrazine (*n*=3).

No.	Hydrazine concentration (ppm)	Remarks
1	0.1	Experiment
2	0.5	"
3	1	"
4	5	"
5	5	Experiment – algae with titanium plate
6	10	Experiment
7	0	Control – algae w/o hydrazine

Table S4: *C. vulgaris* growth with 1–20 ppm hydrazine.

No.	samples name	Hydrazine concentration (ppm)*	Remarks
1	1 ppm	1	Experiment
2	5 ppm	5	"
3	5 ppm +Ti	5	"
4	10 ppm	10	"
5	20 ppm	20	"
6	0 ppm	0	Control – algae w/o hydrazine
7	Br 1	1	Control – Bristol x2 medium with hydrazine
8	Br 5	5	"
9	Br 5 +Ti	5	Control – Bristol x2 + Ti with hydrazine
10	Br 10	10	Control – Bristol x2 with hydrazine
11	Br 20	20	"
12	Br 0	0	Control – Bristol x2 w/o hydrazine

* Hydrazine concentration measurements in algal cultures were taken at T_0 and T_{30}.

Table S5: *C. vulgaris* growth with 20 ppm hydrazine using cultures grown previously (Table S4, No. 1–5). The fresh culture was split to three: 1) FA - same initial OD as reused cultures; 2) FA x2: double initial density; 3) FA/2: half density of FA (No. 7–9). Hydrazine concentration and algal growth were measured in duplicate.

No.	sample name	Hydrazine concentration (ppm)*	Remarks
			Algal source
1	A1	20	Previous experiment –grown with 1 ppm hydrazine
2	A5	20	Previous experiment - grown with 5 ppm hydrazine
3	A5 Ti	20	Previous experiment - with 5 ppm hydrazine + Ti
4	A10	20	Previous experiment - with 10 ppm hydrazine
5	A20	20	Previous experiment - with 20 ppm hydrazine
6	A0	0	Previous experiment control - w/o hydrazine
7	FA	20	Fresh algal culture with OD similar to No. 1–6
8	FA x2	20	Fresh algal culture with 2× density than No. 1–7
9	FA /2	20	Fresh algal culture with half density of No. 1–7
10	Br	20	Control – Bristol x2 medium (w/o algae)
11	Br + Ti	20	Control – Bristol x2 medium + Ti (w/o algae)
12	Br 0	0	Blank – Bristol x2 medium (w/o hydrazine, algae)

* Hydrazine concentrations were measured during the first 24 h, at T_0, T_3, T_6 and T_{24}.

Table S6: Liquid mixed growth media experiment setup for *C. vulgaris* and ISO-36 with 24 well plates in triplicate. Growth was checked at 680 and 600 nm with a plate reader (i-control Infinite 200 PRO, Tecan, Switzerland).

Well number	A1–3	B1–3	C1–3	D1–3	A4–6	B4–6	C4–6	D4–6
Media composition	Bristol X2 100%	Bristol X2 80% +LB 20%	LB only 100%	Bristol X2 100%	Bristol X2 80% +LB 20%	Bristol X2 80% +LB 20%	LB only 100%	Bristol X2 80% +LB 20%
Biological component	-	-	-	*C. vulgaris* OD 0.25	*C. vulgaris* OD 0.25	ISO-36 OD 0.08	ISO-36 OD 0.08	*C. vulgaris* ISO-36

FIGURES

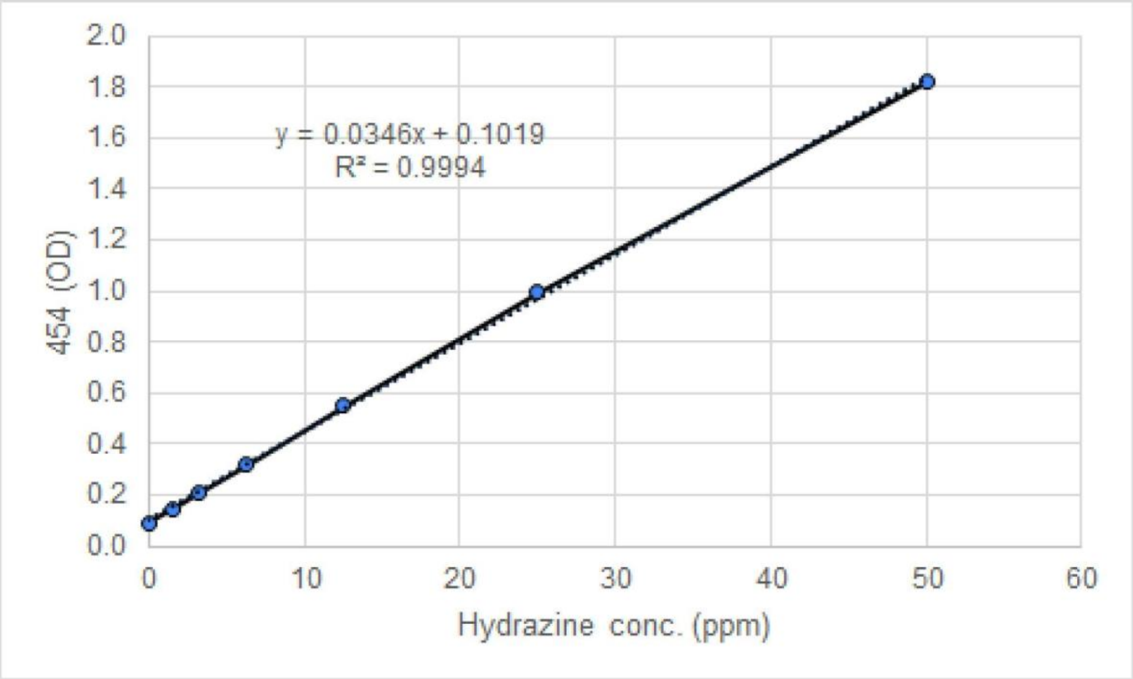

Figure S1: Hydrazine concentration measured at 352 and 454 nm following Gojon and Dureault (1996) with pDMAB 1:50 and six hydrazine concentrations: 0, 2.5, 5, 10, 25 and 50 ppm. Final calibration at 454 nm for hydrazine measurements performed in LB.

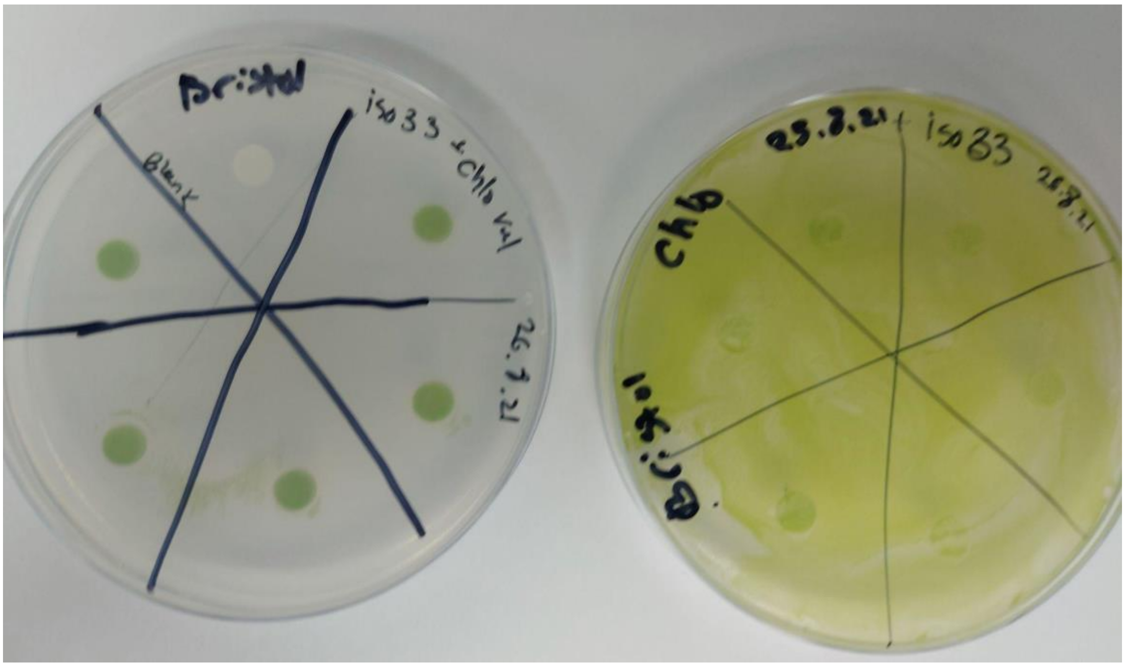

Figure S2. Photo of solid co-culture assessment for isolate ISO-33 and *C. vulgaris,* placed on Bristol agar plates using Whatman assay disks (6 mm) for several days.

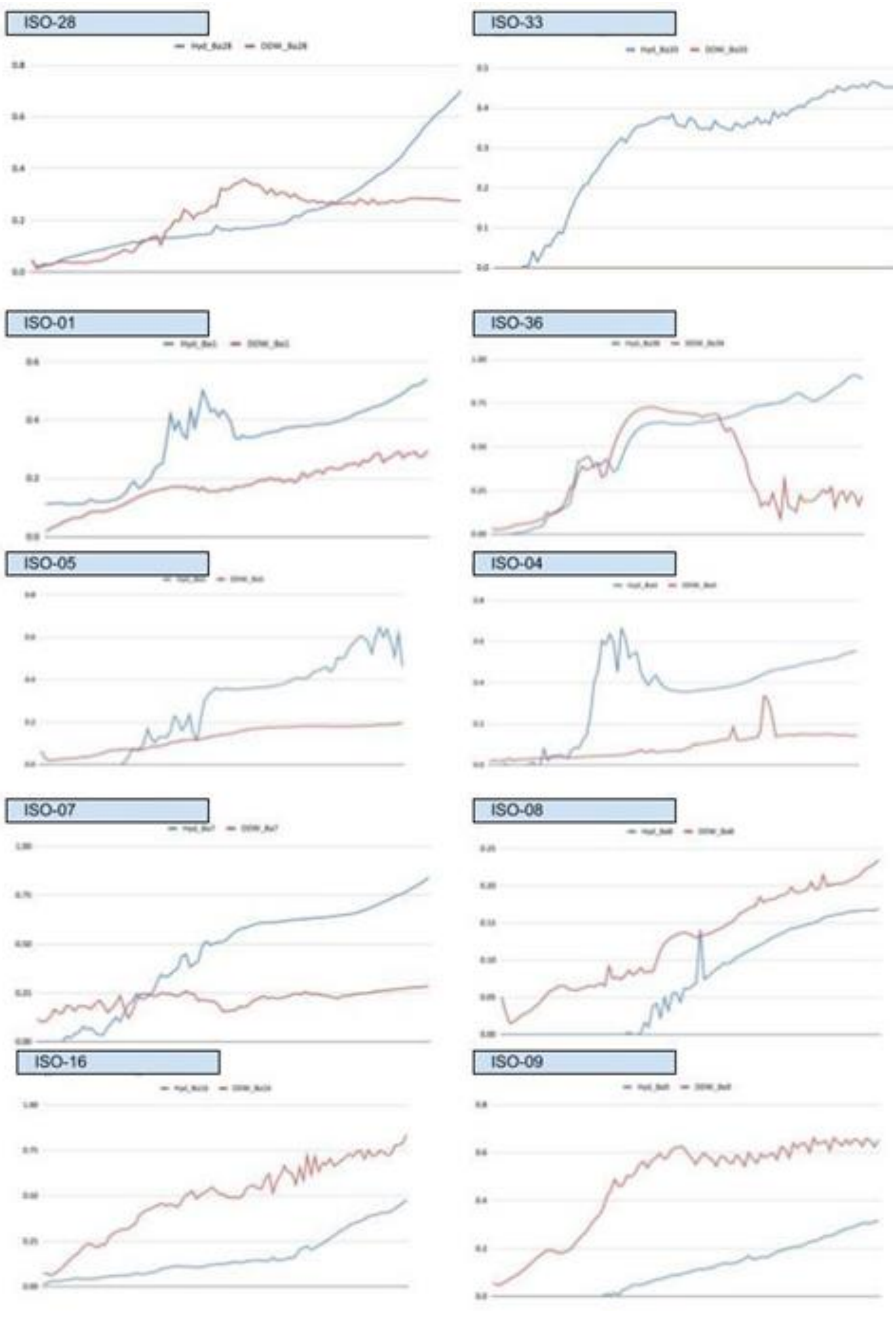

Figure S3: Growth curves of selected bacterial isolates with and w/o hydrazine (25 ppm, blue and red lines, respectively). The y-axis shows OD values measured at different time points (x-axis, 24 h).

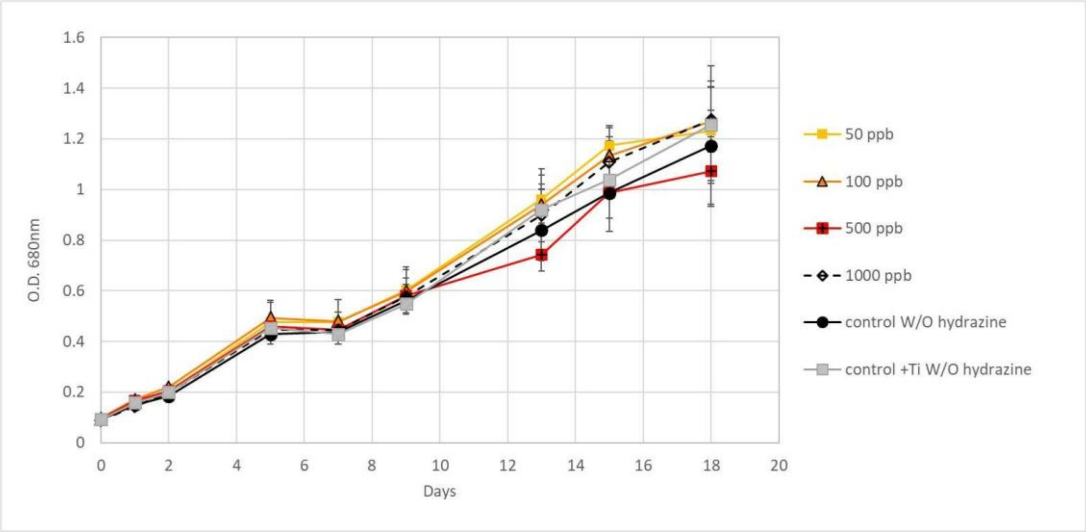

Figure S4: *C. vulgaris* growth graph with 50, 100, 500, 1000 ppb hydrazine and with titanium w/o hydrazine, over an 18-day experiment (*n*=3; with standard deviation).

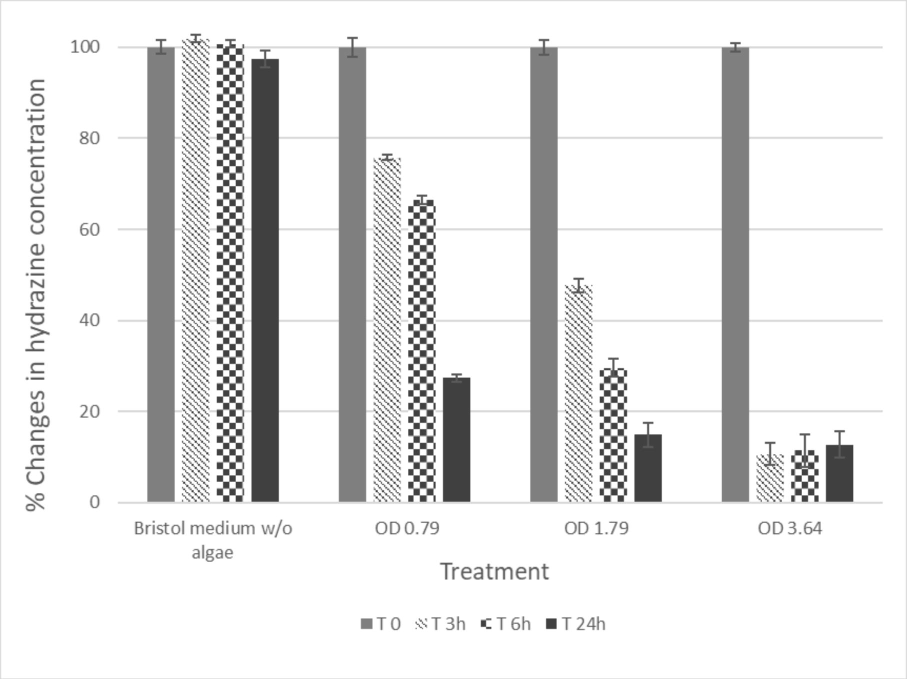

Figure S5: Reductions in hydrazine concentration of algae cultures at different initial density (0.79, 1.79, 3.64 at OD 680 nm) with 20 ppm hydrazine measured at 0, 3, 6 and 24 h. Bristol medium with hydrazine 20 ppm w/o algae was used as control (experiments and measurements were performed in duplicate; error bars are STDEV).

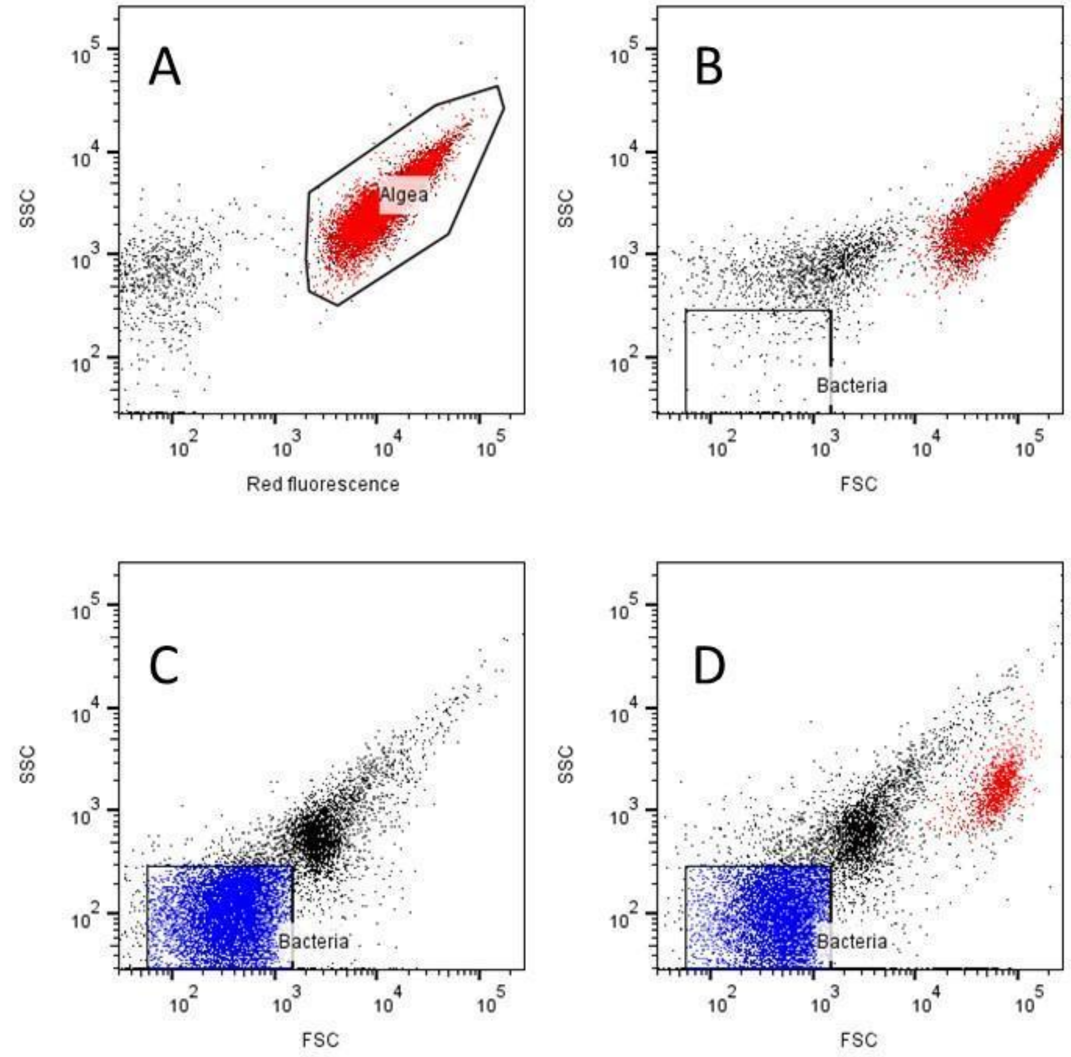

Figure S6: Flow cytometry analysis of algae and bacteria: SSC *vs.* (A) red fluorescence and (B) FSC of an algae-only sample, and SSC *vs.* FSC of (C) a bacteria-only sample and (D) algae and bacteria after 12 h incubation. Algal cells were easily gated owing to auto-fluorescence in the 670 nm channel on the red laser (640 nm).

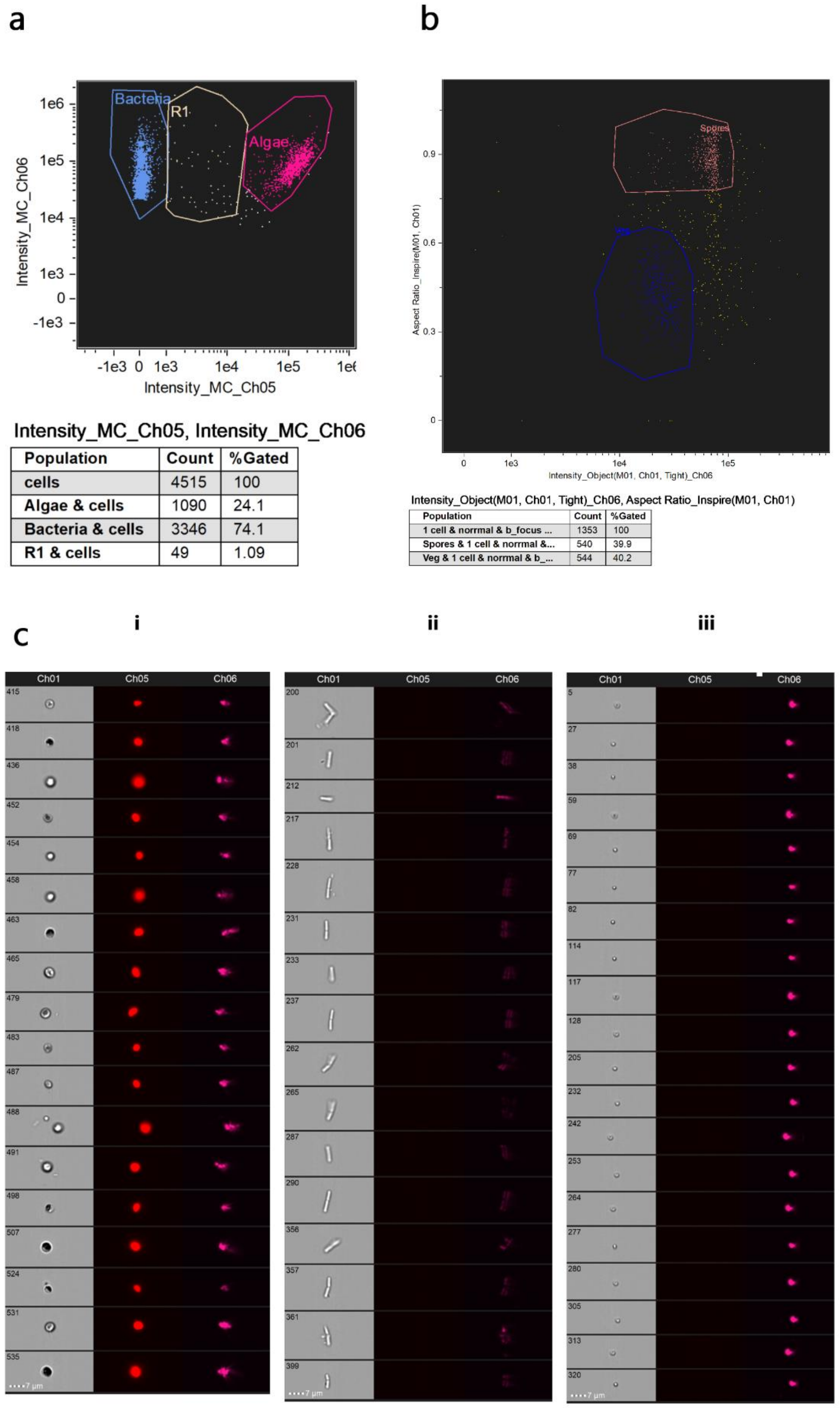

Figure S7:. Imaging flow cytometry analysis of 12-hour incubation experiment: (a) algae and bacteria cells as well as R1 (broken particles) were gated. **(b)** The bacterial population was separated into two types: vegetative and spores. **(c)** Representative images of culture population in channels 01, 05 and 06: (i) algae, (ii) vegetative bacteria, (iii) spores.

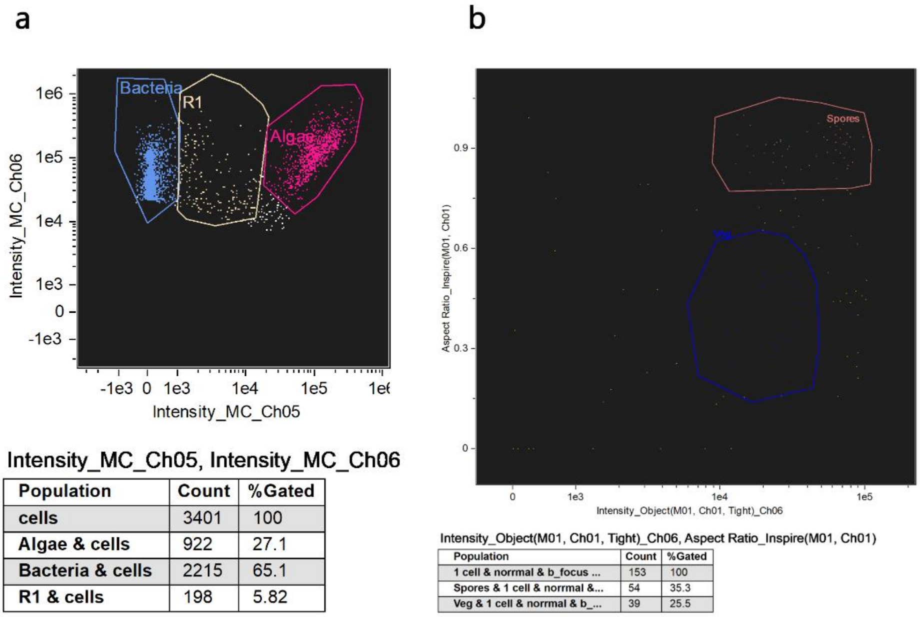

Intensity_MC_Ch05, Intensity_MC_Ch06

Population	Count	%Gated
cells	3401	100
Algae & cells	922	27.1
Bacteria & cells	2215	65.1
R1 & cells	198	5.82

Intensity_Object(M01, Ch01, Tight)_Ch06, Aspect Ratio_Inspire(M01, Ch01)

Population	Count	%Gated
1 cell & norrmal & b_focus ...	153	100
Spores & 1 cell & norrmal &...	54	35.3
Veg & 1 cell & norrmal & b_...	39	25.5

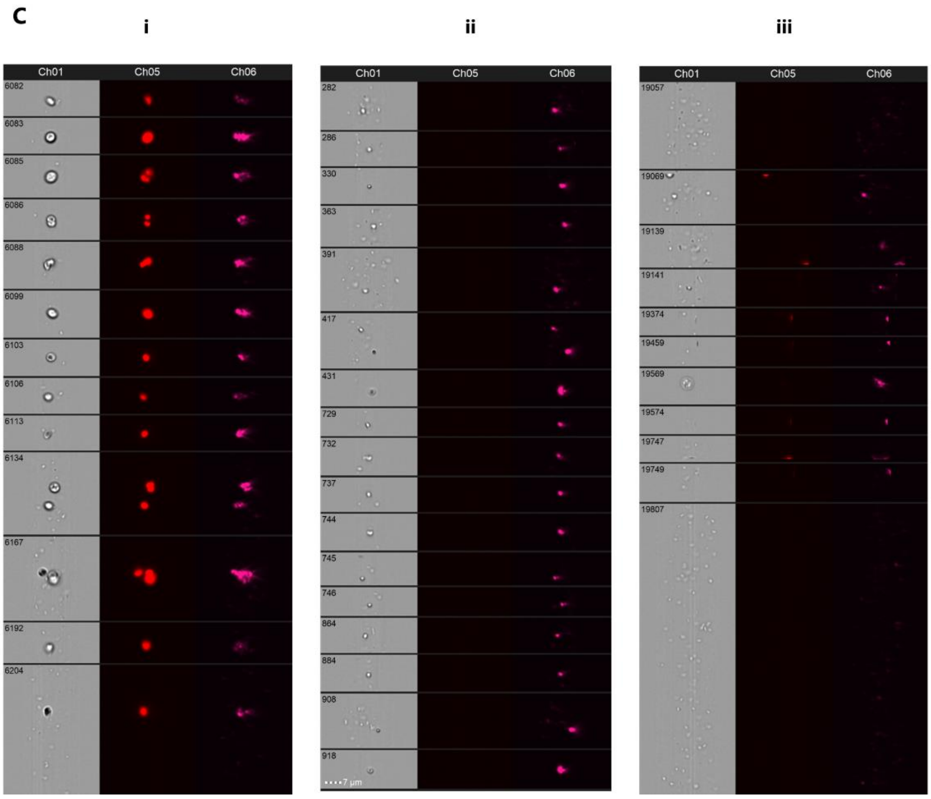

Figure S8: Imaging flow cytometry analysis after 16 day incubation. **(a)** Algae and bacterial cells as well as R1 (broken particles) were gated. **(b)** The bacteria population was separated into two types: vegetative and spores. **(c)** Representative images of culture population in channels 01, 05 and 06: (I) algae, (II) bacteria spores, (III) R1.

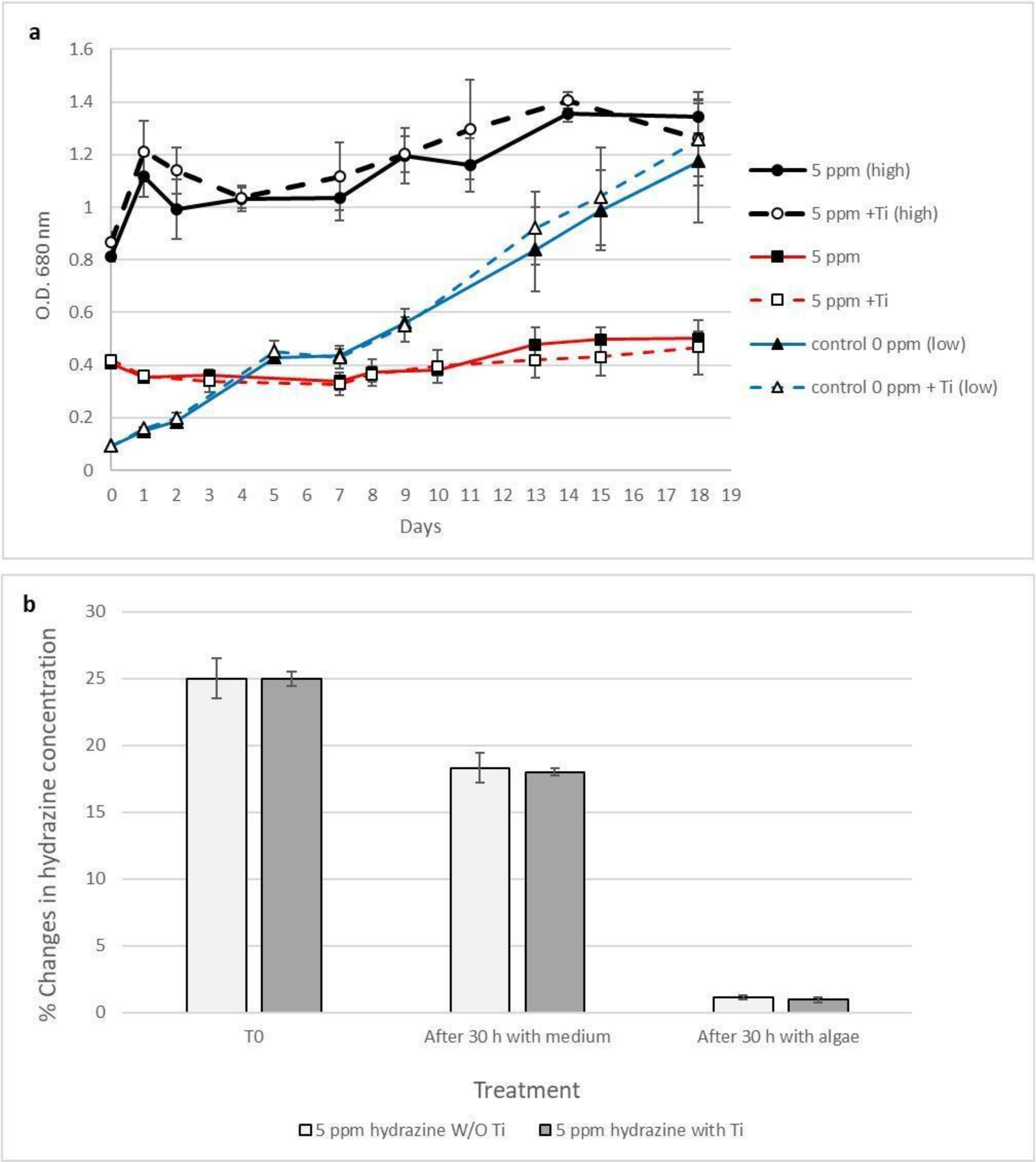

Figure S9: (**a**) *C. vulgaris* growth at different initial culture densities with 5 ppm hydrazine with and w/o titanium (controls: algae w/o hydrazine). (**b**) Hydrazine percentage at T0 and T30 hours (5 ppm=25%, control: Bristol x2 medium, *n*=3, STDEV).

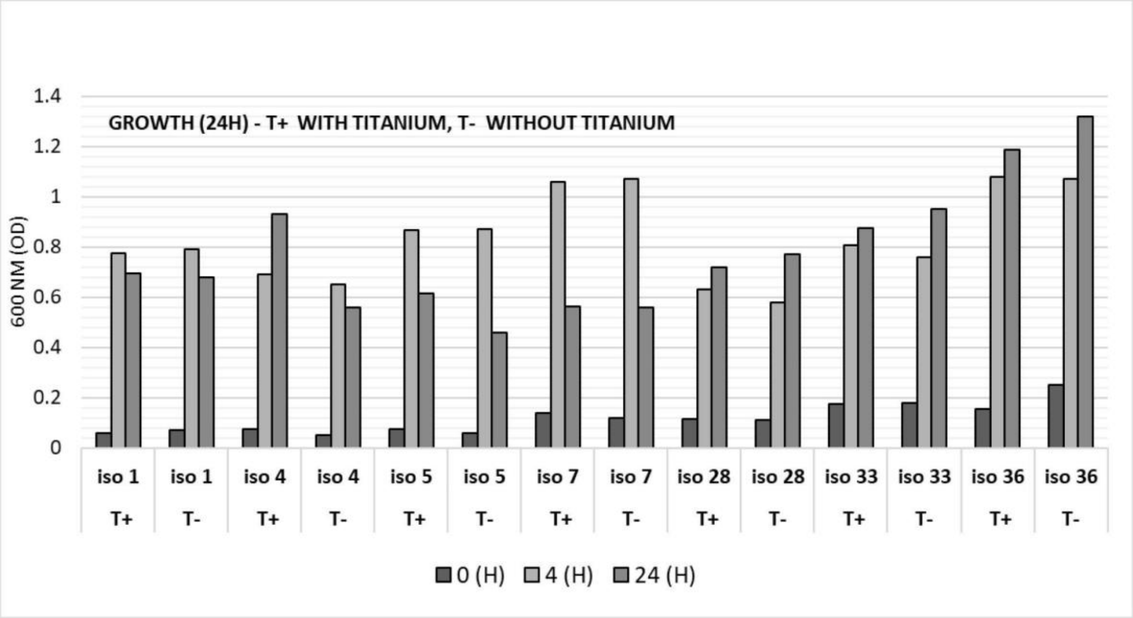

Figure S10: Growth of selected isolates ISO-1/4/5/7/28/33/36 with (T+) and w/o (T-) titanium in LB media at 37° measured at 600 nm at three time points: 0, 4 and 24 h.
